# Robustness of the rule‐learning effect in 7‐month‐old infants: A close, multicenter replication of Marcus et al. (1999)

**DOI:** 10.1111/desc.13244

**Published:** 2022-02-24

**Authors:** Andreea Geambașu, Sybren Spit, Daan van Renswoude, Elma Blom, Paula J.P.M. Fikkert, Sabine Hunnius, Caroline C.M.M. Junge, Josje Verhagen, Ingmar Visser, Frank Wijnen, Clara C. Levelt

**Affiliations:** ^1^ Leiden University Leiden The Netherlands; ^2^ Utrecht University Utrecht The Netherlands; ^3^ AcqVA Aurora UiT The Arctic University of Norway Tromsø Norway; ^4^ Radboud University Nijmegen The Netherlands; ^5^ University of Amsterdam Amsterdam The Netherlands

**Keywords:** artificial grammar learning, language acquisition, replication, rule learning

## Abstract

We conducted a close replication of the seminal work by Marcus and colleagues from 1999, which showed that after a brief auditory exposure phase, 7‐month‐old infants were able to learn and generalize a rule to novel syllables not previously present in the exposure phase. This work became the foundation for the theoretical framework by which we assume that infants are able to learn abstract representations and generalize linguistic rules. While some extensions on the original work have shown evidence of rule learning, the outcomes are mixed, and an exact replication of Marcus et al.'s study has thus far not been reported. A recent meta‐analysis by Rabagliati and colleagues brings to light that the rule‐learning effect depends on stimulus type (e.g., meaningfulness, speech vs. nonspeech) and is not as robust as often assumed. In light of the theoretical importance of the issue at stake, it is appropriate and necessary to assess the replicability and robustness of Marcus et al.'s findings. Here we have undertaken a replication across four labs with a large sample of 7‐month‐old infants (*N *= 96), using the same exposure patterns (ABA and ABB), methodology (Headturn Preference Paradigm), and original stimuli. As in the original study, we tested the hypothesis that infants are able to learn abstract “algebraic” rules and apply them to novel input. Our results did not replicate the original findings: infants showed no difference in looking time between test patterns consistent or inconsistent with the familiarization pattern they were exposed to.

## Graphical abstract

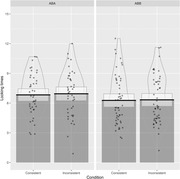

## 1 INTRODUCTION

In a seminal, often‐cited paper in the journal *Science*, Marcus et al. (1999) presented three experiments suggesting that infants as young as 7 months are able to do something quite extraordinary: after hearing a speech stream consisting of syllables arranged in an ABA, ABB, or AAB pattern, they detect (and learn) the underlying pattern and can apply it to syllable sequences they have not heard before. After brief exposure to trisyllabic strings that instantiated one of the patterns, infants showed more interest (i.e., longer looking times) during the test phase to novel syllables arranged in a novel order, compared to novel syllables arranged in the familiar order. The authors interpreted these results as evidence of infants’ ability to extract algebraic rules from a speech stream. It may seem a simple task to do, yet the ability to abstract away from the syllables presented to form abstract categories or variables, had never been shown in such young infants before. The literature at the time had only shown that infants of this age are sensitive to distributional patterns and can use them to segment words from speech streams (Saffran et al., 1996). The work of Marcus et al. took our understanding of what infants can do at such an early age one step further, showing that, in addition to using statistical regularities, infants are also sensitive to abstract regularities, a cognitive ability that is thought to be at the basis of learning the rules of language (grammar or syntax), and therefore potentially the very foundation of what sets humans apart from other species.

In the years that followed, several studies attempted to extend these findings with different age groups and in different domains (e.g., Gerken, 2006; Marcus et al., 2007; Rabagliati et al., 2012; Saffran et al., 2007), with adults (e.g., Christiansen et al., 2000; Geambașu, 2018), and with non‐human species (e.g., Chen et al., 2015; Spierings & Ten Cate, 2016). Overall, these replications differed from the original study in one or more key aspects and yielded mixed results. However, as no exact replication of the study of Marcus et al. (1999) has been published to date, it is still unclear whether the original outcome can be replicated, and with what magnitude of an effect. The original study has had far‐reaching theoretical implications, and as such, it is essential that we reassess its empirical basis. With an increased attention placed on replication in the past years, and given the immense importance of this study, the present work focuses on attempting to replicate the work of Marcus et al. (1999) as closely as possible.

### 1.1 Previous extensions and near‐replications

Since the publication of the original paper by Marcus et al. (1999), the same rule learning paradigm has been used by researchers studying the capacity for abstraction—and its limits—in infants, adults, and non‐human species. In the first published extension, Gerken (2006) used a subset of the original stimuli (four familiarization items instead of 16) in order to investigate whether infants are able to abstract a rule from fewer exposure items and the impact of the amount of variety within the exposure set. Infants were only able to abstract a rule if the ABA‐type rules were constructed from four items composed of completely different syllables (e.g., *ledile, wijewi, jiliji, dewede*) and not when the rules were constructed from four items that all had the same “B” items (e.g., *le* ***di*** *le, wi* ***di*** *wi, ji* ***di*** *ji, de* ***di*** *de*). While these results seem to support and build on the original Marcus et al. findings, there were several important differences between the two studies. Gerken's work was conducted with 9‐month‐old infants instead of 7‐month‐olds. A footnote in Gerken (2006) indicates that piloting on AAB versus ABB with 7‐month‐olds using the full set of 16 syllables as in Marcus et al. (1999) failed to replicate the original results, and instead showed a trend towards an overall ABB preference instead. While the older infants were able to abstract the patterns, and were able to do so from the smaller subset of ABA or AAB triads they were exposed to, Gerken's study is an extension, rather than a replication and calls into question the strength of the original paper's results. There are some differences in the procedure that might account for the success of the original study and the failure to replicate it with the same age group in Gerken (2006). As pointed out by Gerken in her original paper, her experiment only used four familiarization items and four test trials, likely leading to a difference in looking behavior during test trials as compared to the original results of Marcus et al. (1999). While Marcus et al. had found a novelty preference during the test, Gerken found a familiarity preference, indicating that infants in Gerken's experiment had not fully finished learning about the familiarization items. While the main idea remained roughly the same—that generalization on the basis of a short exposure was possible in young infants—Gerken's work introduced the notion that various changes in stimuli could produce very different patterns of behavior.

**RESEARCH HIGHLIGHTS**

- Our study offers a large‐scale replication of the high‐impact, seminal study by Marcus et al. (1999) across four labs.
- Using the same materials as in the original study, we did not find evidence of rule learning.
- The study enabled us to better evaluate the robustness of the algebraic rule learning effect.
- A better understanding of rule‐learning abilities in infants has an impact on theories related to general cognitive abilities of humans and other animals.

Since that first notable extension, many others have been conducted, both in the linguistic (Gerken et al., 2015) and in other domains, including studies with sign language stimuli (Rabagliati et al., 2012), nonlinguistic auditory stimuli (Ferguson & Lew‐Williams, 2016; Marcus et al., 2007), and nonlinguistic visual stimuli (Johnson et al., 2009; Saffran et al., 2007). Such studies have had mixed results, with success of rule learning found to be tied to age, stimulus type, salience, surprise level, and communicative value of the stimulus. Rabagliati et al. (2019) meta‐analyzed the effects of more than 60 such studies, both published and unpublished, on rule learning across different age groups (4–13 months) and domains. While the analysis found evidence for an overall novelty effect, indicating the ability to learn abstract rules in infants, the authors also found a great deal of variability between results and effect sizes, even though most studies were sufficiently powered. The mixed results of this meta‐analysis, and the studies on which it focuses, emphasize that there are outstanding questions that must be answered before we may have a clear picture of when (i.e., at what age) and how (i.e., using which types of stimuli, conditions, tasks) rule learning works. Finally, and worryingly, the authors found indications of publication bias in the literature surrounding this phenomenon, raising the possibility that unsuccessful replication attempts suffer from the so‐called file‐drawer problem and driving the view that rule learning is a closed case. A search for clarification of outstanding issues must start with the original effect.

Geambașu and colleagues have a number of results that speak to this issue. In a study in their lab in the Netherlands, using both nonspeech auditory stimuli (birdsong) and naturally recorded Dutch syllables and a visual fixation paradigm with 6‐ and 9‐month‐old infants, they found no difference in discrimination abilities of infants as a function of familiarization pattern (Geambașu, 2018). A second near‐replication, in which stimuli, age group, and procedures were kept very similar to the ones used in Experiment 2 of Marcus et al.’s original work (testing 7‐month‐olds using synthetic—Dutch—syllables and the Headturn Preference Paradigm, as in the original), was also unsuccessful (Geambașu, 2018). Across all of these experiments, infants instead showed a strong preference for repetition patterns (AAB or ABB) regardless of which familiarization pattern they were exposed to (AAB, ABB, or ABA; Geambașu, 2018). Infants in these previous studies seem to show a preference for test trials containing repetition. This pattern of results indicates that Dutch infants may have a general bias for listening to repetition patterns. This does not, however, necessarily translate to more or less learning from those patterns—see the Analysis section for more discussion on this point.

If differences in stimuli, experiment setup, and background of the participant population indeed result in an inability to replicate the original findings, it seems justified to evaluate the robustness of the effect originally reported, and add to the nuance in the discussion about *if* and *when* generalization can take place (Rabagliati et al., 2019; Schonberg et al., 2018).

### 1.2 Current study

The findings of Marcus et al. speak to what should be a general ability of all infants in any language background. Yet previous attempts at replication and the mixed results that have come from the various extensions leave us with many unanswered questions about the details of when and how rule‐learning takes place. In the present work, we aimed to replicate as closely as possible the original work on a larger scale in order to be more certain of the robustness of the original results. To this end, we replicated the study with 96 participants distributed over four labs in the Netherlands.

We are aware of (and some of our authors are involved in) even larger effort to replicate Marcus et al. (1999) as part of the ManyBabies 3 project (MB3). However, we differentiated ourselves from that replication effort in several important ways, related both to the goals and the implementation of the studies. The goal of the present study was to remain as faithful as possible to the original work and to answer the original research question: whether infants of 7 months old are able to extract and generalize abstract, algebraic rules from an artificial speech stream. As such, all methods, procedures, and stimuli were maximally similar to the original study. In contrast, the goal of MB3 is to find out under which conditions rule learning proceeds best; between‐lab modifications to the original methods are made based on international labs’ standard practices and these modifications’ effects on learning will later be evaluated. In addition, a new set of “international” stimuli were made for MB3. There are therefore important differences between our two projects, which render them both necessary and complementary. Both large‐scale replication efforts will allow us to more confidently assess whether the original results are replicable with a larger group of infants of different language backgrounds than the original and will capture potential nuances the original study was not able to capture with the smaller group of participants (*n* = 16).

Here, we tested the original hypothesis that infants are able to learn abstract rules. Successful replication would add to the robustness of the original findings by adding evidence from a different, and larger, subject population. Failure to replicate the original results, on the other hand, would lead us to conclude that the original results are not robust and algebraic rule learning may not be as generally available to 7‐month‐olds as has been thought since the publication of Marcus et al. (1999). Such a result can be of two different types: using Bayesian analyses, we will be able to differentiate between a null‐result—evidence that the rule learning effect is negligible in size—and the situation that there is simply a lack of evidence to distinguish between a positive effect size and a null effect size. Given the mixed results present in the literature, and given literature that more consistently seems to point towards the salience of repetition (e.g., Gervain et al., 2008; Gerken et al., 2015; Geambasu, 2018), it is also possible that results may rather support such effects than those found in the original. Either outcome will have a high impact on theories of language acquisition.

## 2 METHODS

### 2.1 Participants

Our final sample included 96 infants (46 girls, 50 boys) who were on average 214 days old (*SD = *9 days, min–max = 196–228 days)^1^. The linguistic background of the participants in the original Marcus et al. (1999) study was not specified. Because of the location of our study, we expected to test mostly monolingual Dutch infants, but did not exclude participants who had a different language background. Caregivers filled out an adaptation of the Language Exposure Questionnaire (LEQ, Bosch & Sebastián‐Gallés, 2001, Cattani et al., 2014), which provides an estimate of children's language exposure, based on the relative amounts (i.e., percentage) of exposure in each language. Eventually, we tested 58 monolingual children (hearing one language more than 90% of the time), 10 unbalanced multilingual children (hearing one of their languages between 90% and 75% of the time), and 28 balanced multilingual children (not hearing one language more than 75% of the time). Infants had no developmental delays, and were full‐term at birth (i.e., had a gestational age of at least 37 weeks, according to the protocols of The Many Babies Consortium (2020). Participants were recruited via the municipalities of Amsterdam, Leiden, Nijmegen, and Utrecht, social media outreach, and personal recruitment efforts and networks.

Data collection took place between February 2021 and December 2021, and continued until the required number of participants was reached, which was determined on the basis of a simulation that can be consulted in the Stage 1 Registered Report, available on our OSF page. We oversampled until we reached the required number of infants. To be able to include the required 96 children in our final sample, we tested 114 children^2^. Children were excluded on the basis of online and offline criteria. Online exclusion criteria were: excessive fussiness or distress/irritability during testing that prevented the infant from attending to the stimuli (*n* = 12); parental interference, technical‐ and/or experimenter errors (*n* = 2); failing to contribute to at least four out of the 12 test trials, or hitting the maximum looking time on eight or more test trials (*n *= 0). Offline exclusion criteria were: having average looking times greater than 2.5 SD above or below the experimental mean (*n *= 1); developmental delays or preterm birth status (*n *= 2); children falling outside the age range (*n* = 1, age = 237 days).

### 2.2 Stimuli

In the original Marcus et al. (1999) study, infants were familiarized and tested with the Headturn Preference Procedure. The infants were exposed to 2‐min auditory familiarization sequences composed of ABA or ABB triads (in Marcus et al. referred to as “sentences”). Subsequently, infants heard novel three‐syllable test sequences that were arranged in triads either consistent or inconsistent with the familiarization pattern. Infants familiarized with either ABA or ABB were tested with both ABA and ABB patterns to assess their preference for test patterns based on their consistency with the familiarization pattern. The original findings showed that infants had longer looking times to inconsistent trials than to consistent trials, that is, they showed a novelty preference during the test phase. The present work followed the same procedures.

We acquired the original auditory stimuli files (as syllables)^3^ and created our familiarization and test items from them. Table 1 shows a detailed list of the syllables, their phonetic transcriptions, which category they were assigned to, and the durations of the syllables and the triads.

**TABLE 1:** Syllables of category A and B and triads (sentences) including orthographic spelling and IPA transcription in brackets and durations in milliseconds

Syllable A	Syllable A duration	Syllable B	Syllable B duration	Syllable A–B difference	Triad duration ABA	Triad duration ABB
le [li]	216.8	di [daɪ]	415.2	−198.4	1348.8	1547.2
wi [waɪ]	411.3	di [daɪ]	415.2	−3.9	1737.8	1741.7
ji [dʒaɪ]	410.6	di [daɪ]	415.2	−4.6	1736.4	1741.0
de [deɪ]	378.0	di [daɪ]	415.2	−37.2	1671.2	1708.4
le [li]	216.8	li [li]	314.8	−98.0	1248.4	1346.4
wi [waɪ]	411.3	li [li]	314.8	96.5	1637.4	1540.9
ji [dʒaɪ]	410.6	li [li]	314.8	95.8	1636.0	1540.2
de [deɪ]	378.0	li [li]	314.8	63.2	1570.8	1507.6
le [li]	216.8	we [wi]	240.8	−24.0	1174.4	1198.4
wi [waɪ]	411.3	we [wi]	240.8	170.5	1563.4	1392.9
ji [dʒaɪ]	410.6	we [wi]	240.8	169.8	1562.0	1392.2
de [deɪ]	378.0	we [wi]	240.8	137.2	1496.8	1359.6
le [li]	216.8	je [dʒi]	342.8	−126.0	1276.4	1402.4
wi [waɪ]	411.3	je [dʒi]	342.8	68.5	1665.4	1596.9
ji [dʒaɪ]	410.6	je [dʒi]	342.8	67.8	1664.0	1596.2
de [deɪ]	378.0	je [dʒi]	342.8	35.2	1598.8	1563.6
**ba [bʌ]**	**397.6**	**po [pʊ]**	**331.8**	**65.8**	**1627.0**	**1561.2**
**ko [kʊ]**	**346.0**	**ga [gʌ]**	**273.8**	**72.2**	**1465.8**	**1393.6**

*Note*: The final two rows, in bold text, were the items used during the test phase, while the rest were used exclusively in the familiarization phase. Triad duration ABA = (2*duration syllable A) + duration syllable B + (2*250‐ms intersyllable pause; triad duration ABB = (2*duration syllable B) + duration syllable A + (2*250‐ms intersyllable pause).

The familiarization streams resulting from the combination of syllables consisted of 16 different triads, with each triad separated by a 1‐s pause. The familiarization streams were created from a custom Python script that concatenated the triads in a random order in three “blocks” per stream, to form a 2‐min stream with three sequential random presentations. Sixteen of these unique files were created from the familiarization triads that are listed in Table 2.

**TABLE 2:** Syllables arranged into ABA and ABB familiarization triads and test triads. Familiarization triads following either the ABA or ABB pattern were presented in random order, with familiarization pattern counterbalanced across participants; all participants heard both ABA and ABB test triads

Familiarization triads			
ABA		ABB	
le di le	ji di ji	le di di	ji di di
le je le	ji je ji	le je je	ji je je
le li le	ji li ji	le li li	ji li li
le we le	ji we ji	le we we	ji we we
wi di wi	de di de	wi di di	de di di
wi je wi	de je de	wi je je	de je je
wi li wi	de li de	wi li li	de li li
wi we wi	de we de	wi we we	de we we

Test triads			
ABA		ABB	
ba po ba	ko ga ko	ba po po	ko ga ga

The familiarization phase was followed by a test phase composed of four different test triads, two following the ABA pattern and two following the ABB pattern. Each test trial consisted of one triad repeated six times, with each repetition separated by a 1‐s pause. The test trials were repeated across three blocks, and randomized per block, resulting in 12 total test trials. As listed in Marcus et al., the test trials consistent with the ABA pattern were “ba po ba” and “ko ga ko” while the test trials consistent with the ABB pattern were “ba po po” and “ko ga ga.” Table 2 shows a list of both familiarization and test triads.

### 2.3 Procedure—general protocols

The general welcome and set up procedures can be viewed at the ManyBabies video repository at https://nyu.databrary.org/volume/896 for three of our four labs (Leiden, Nijmegen, and Utrecht). The procedures at the Amsterdam lab did not deviate in any meaningful way from those in the other labs. Efforts were made to be as consistent as possible across labs with respect to general procedures, while at the same time, allowing the typically practiced technical protocols of each lab to continue as normal. This meant that the order of operations during the procedures was as similar as possible (with regards to welcoming, filling out questionnaires, etc.), while differences were present in stimulus presentation software, technical specifications of hardware, and experimental space.

In all cases, caregivers were contacted and informed about the study prior to making an appointment to come to the lab. Infants and their caregivers were welcomed to the lab by the experimenter, were informed again in more detail about the goal and procedure of the study via a written document and orally about the importance of remaining calm and holding their infant comfortably while not influencing or reacting to them (only during the attention grabber, unless the infants were in distress), and about their rights as participants to stop the experiment and to withdraw consent. Before beginning the experiment, caregivers signed an informed consent form indicating that they consent to their infant's data to be used for research purposes. The research had been assessed and approved by the ethics committees of each university. This was communicated to the caregivers, and they were provided with contact information in the event that they wished to lodge a complaint with the respective universities.

After consent was obtained, caregivers and infants were led to the experimental area, where the experiment proceeded. Caregivers were provided headphones playing a mix of music and speech in order to mask the auditory stimuli of the experiment. This prevented them from reacting and unwittingly influencing their infant's behavior. Infants sat on their caregiver's lap for the duration of the experiment. After the experiment ended, caregivers and infants were led out of the experimental area (a booth, a separate room, or a separate section of the same room, depending on the lab) and back to the welcome area. They were then asked to fill out a questionnaire about their infant and their family background if they had not done so in advance, and were compensated for their participation (depending on the lab, either in the form of reimbursed travel costs, a children's book, or cash).

### 2.4 Procedure—experimental design

Infants took part in a between‐subject experiment in which they were randomly assigned to one of the two conditions, ABA or ABB condition, in which they were familiarized with triads following an ABA or ABB pattern accordingly (as in the original study's Experiment 2). As in the original study, the experiment was carried out using the Headturn Preference Procedure. In this procedure, infants sit on their caregiver's lap in a quiet room, while an experimenter who observes the infant's behavior and controls the experiment sits outside of the experimental area, hidden from the participants’ view.

The procedure was composed of a familiarization phase of fixed duration and a test phase. In the familiarization phase, an attention grabber light (or screen) first flashed directly in front of the participants. Once infants had directed their gaze to the attention grabber, an auditory familiarization stimulus stream played for 2 min from speakers located (in front or) on both sides of the infant, while the attention grabber light continued to flash. Both the visual and the auditory familiarization stimuli played continuously and independently of the infant's behavior.

After the familiarization stream ended, the test phase started. Every test trial started with the attention grabber playing until the infants looked towards it. Once the experimenter indicated that the infant had refixated onto the attention grabber, one of two sidelights/screens, either on the left or the right side of the infant, began to flash. When the infant looked towards the side light/screen, the test stimulus began to play from a speaker located in close proximity to the light/screen (either underneath, above, behind, or in front), and continued to play for as long as the infant looked in the direction of the light/screen. If the infant looked away for less than two continuous seconds, the light/screen continued to flash and the auditory stimulus continued to play. If the infant looked away for more than two continuous seconds, both the visual and auditory stimulus ended, and thus the test trial ended. Each test trial repeated the same auditory stimulus for a maximum of 15 s, which equals six triads per trial. This experimental procedure was repeated until the infants completed the test phase, or until either the caregiver or experimenter indicated that the infant was too fussy (not orienting to attention grabber for an extended period of time) or distressed (crying or screaming while ignoring the lights) to continue.

The test phase consisted of four test trials composed of syllables not used during the familiarization, two of which followed a pattern inconsistent with the familiarization, and two of which followed a pattern consistent with the familiarization. These four test items were repeated for three blocks, and were randomized per block, totaling 12 test trials with a maximum duration of 15 s each.

To ensure that the infants were not influenced in any way during the experiment, neither participants’ caregivers nor experimenters were aware of which test items infants were presented with during the test. However, for the sake of counterbalancing (ensuring that an equal number of participants are exposed to ABA and ABB learning phases), the experimenter did know to which familiarization condition the infants were assigned.

After analysis of three pilot videos by two coders, we found that offline and online coding correlated with 97% accuracy and intercoder reliability correlated with 94% accuracy. We, therefore, decided to use the online coding output as our dependent variable in the experimental analysis.

## 3 ANALYSES

All analyses were carried out in R (R Core Team, 2015). Scripts and data from these analyses are available on our OSF page. The consistency of the test pattern as compared to the familiarization pattern (consistent or inconsistent) was the explanatory variable, while the behavioral response (looking times in milliseconds) was the outcome variable. This is identical to the original study. First, we analyzed the data using the same methods as in the original Marcus et al. (1999) study, that is, by applying a repeated‐measures ANOVA to the looking times in the test phase of the experiment, comparing looking times to test stimuli consistent or inconsistent with the familiarization pattern. In addition, we conducted a Bayesian analysis, which was not conducted in the original study. The added value of such an analysis is that the Bayes factor can differentiate between evidence for the null, evidence for the alternative, and lack of evidence altogether. A BF indicates the ratio of the likelihood of a given hypothesis compared to some other hypothesis (Beard et al., 2016). A BF_10_ of 10, for example, indicates that it is 10 times more likely that the data would be observed under the alternative hypothesis than under the null hypothesis. A BF_01_ of 3, on the contrary, indicates that it is three times more likely that the data would be observed under the null hypothesis than under the alternative hypothesis. A BF between 10 and 30 might be seen as strong evidence, a BF of 3–10 as moderate evidence, whereas a BF between 1 and 3 merely provides anecdotal evidence for a particular hypothesis (Lee & Wagenmakers, 2013).

The Bayesian analysis also included effects of trial number and repetition (Endress et al., 2009; Geambașu, 2018; Gervain et al., 2008) that are likely to influence the looking times. The model also included random intercepts for trial type (i.e., the four test sentences), participant, and lab, with the random factor for participant nested in the one for lab, since certain groups of participants were tested in a particular lab. Furthermore, we estimated random slopes for trial number and consistency per participant, since these are within‐participant fixed effects, as well as slopes for familiarization pattern and trial number per trial type, since these are within‐item fixed effects. We used the R‐package *brms* (Bürkner, 2021) to fit the following model, in which we included generic weakly informative priors for all fixed effects (Gelman et al., 2008; Gelman, 2020): $\begin{array}{c} \mathrm{LT}∼\;\;\;\;1+\mathrm{trial}\_\mathrm{number}+\mathrm{familiarization}\_\mathrm{pattern}+\mathrm{consistency}+\\ \;\;\;(1+\mathrm{trial}\_\mathrm{number}+\mathrm{consistency}|\mathrm{PP}/\mathrm{LAB})+\\ \;\;\;(1+\mathrm{trial}\_\mathrm{number}+\mathrm{familiarization}\_\mathrm{pattern}|\mathrm{trial}\_\mathrm{type}) \end{array}$

In this model, looking time was modeled as a function of the trial number, trial type, and consistency. In general, if children learned the pattern in the input, we would expect a main effect of consistency: looking times should be longer for test trials that are inconsistent with the input from the familiarization phase than for trials that are consistent with the input from the familiarization phase. This main effect was also observed in the original study. However, we might also expect an interaction between familiarization pattern and consistency of the test sentences in our experiment, which was not observed in the original study. Specifically, we could imagine that a bigger difference between consistent and inconsistent trials would be observed in the ABB familiarization condition: strings containing a syllable repetition might be less surprising for young children than strings that do not contain such a repetition (Gerken et al., 2015), for example, because Dutch children tend to produce such strings more (Geambașu et al., 2016). If such a surprise bias is indeed present, this should lead to a bigger difference between consistent and inconsistent trials in the ABB familiarization condition than in the ABA familiarization condition. On the other hand, if children did not learn either of the two patterns, but instead had a general repetition preference (Geambașu, 2018), this bias would lead to a preference for inconsistent over consistent test trials in the ABA familiarization condition, and a preference for consistent test trials in the ABB familiarization condition. In other words, if children did not learn the patterns, but showed evidence of a repetition bias, we should also observe an interaction, but not a main effect of learning. In addition, we expected that looking behavior in the first block after familiarization might be a better indicator of learning than looking behavior across all blocks. In line with the above comment, discrimination patterns in the first block (trials 1–4) but not in the second and third blocks (trials 5–12) would be interpreted as evidence of learning. As an exploratory analysis, and to test such effects of trial during the experiment, we ran the same mixed‐effects model as described above, but now with all possible interaction effects included both for our fixed effects and random slopes.

## 4 RESULTS

**FIGURE 1:**
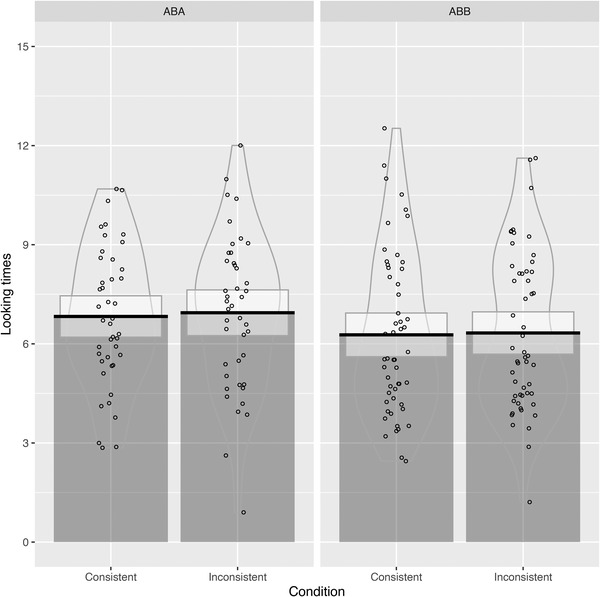
Pirate plots visualizing the results from our head‐turn experiment for all labs combined, split by the type of familiarization pattern infants were exposed to in the input

**FIGURE 2:**
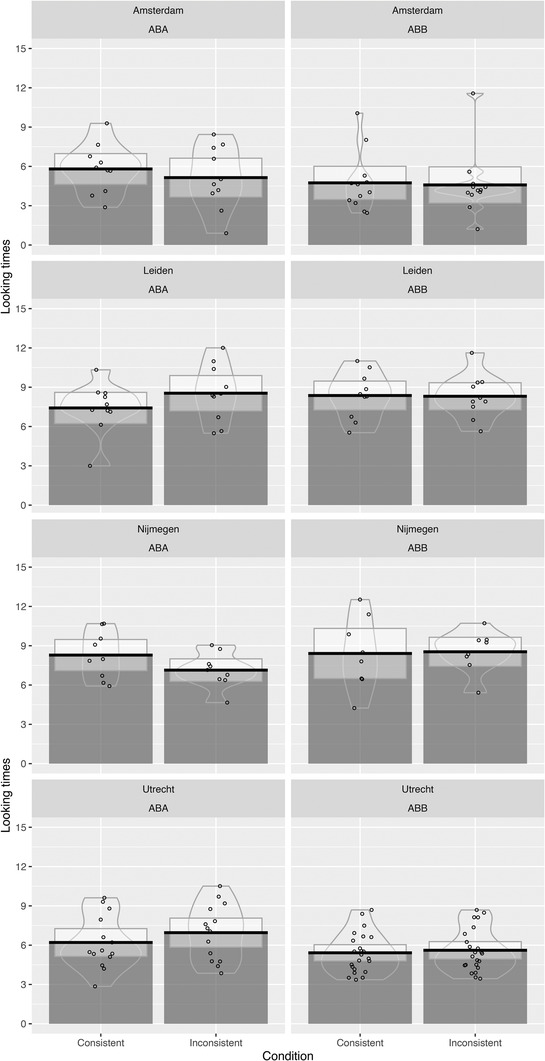
Pirate plots visualizing the results of the head‐turn experiment for all labs separately

Table 3 shows descriptives of the looking times per familiarization pattern and test sentence consistency, which are visualized in Figure 1 (all labs together) and Figure 2 (split out per lab).

**TABLE 3:** Descriptives for the looking times that were observed in the head‐turn experiment for both experimental conditions

		Consistent			Inconsistent		
	*n*	*M*	*SD*	min–max	*M*	*SD*	min–max
*All labs*	96	6.52	2.30	2.45…12.52	6.60	2.35	0.9…11.11
*Amsterdam*	22	5.22	2.11	2.45…10.06	4.84	2.38	0.9…11.57
*Leiden*	20	7.89	1.87	3.00…11.00	8.42	1.89	5.49…12.00
*Nijmegen*	17	8.35	2.23	4.24…12.52	7.80	1.58	4.66…10.72
*Utrecht*	37	5.72	1.74	2.85…9.61	6.12	1.91	3.44…10.51

*Note*: Looking times were measured in full seconds. We presented average looking times for the two conditions combined, since we did not observe an effect of familiarization pattern. Descriptives for the two conditions separately can be consulted in supplementary materials, and are depicted in Figures 1 and 2.

We first analyzed these data using the same types of ANOVAs as in the original study. Our two‐way ANOVA with looking times as a dependent variable, and familiarization pattern and consistency as independent variables showed no significant main effect of test sentence consistency on looking times (*F*(1, 94) = 0.155, *p *= 0.695): children did not look significantly longer at trials that were inconsistent with the input from the familiarization phase compared to trials that were consistent with this input. We also did not observe a main effect of familiarization pattern (*F*(1, 94) = 1.881, *p *= 0.174), nor an interaction between familiarization pattern and trial type (*F*(1, 94) = 0.017, *p *= 0.896), which would have been indicative of a repetition bias. A second ANOVA, which only included consistency as an independent variable, but not familiarization pattern, which was conducted in the original study, did not show a significant effect of test sentence consistency either (*F*(1, 95) = 0.148, *p *= 0.702).

**FIGURE 3:**
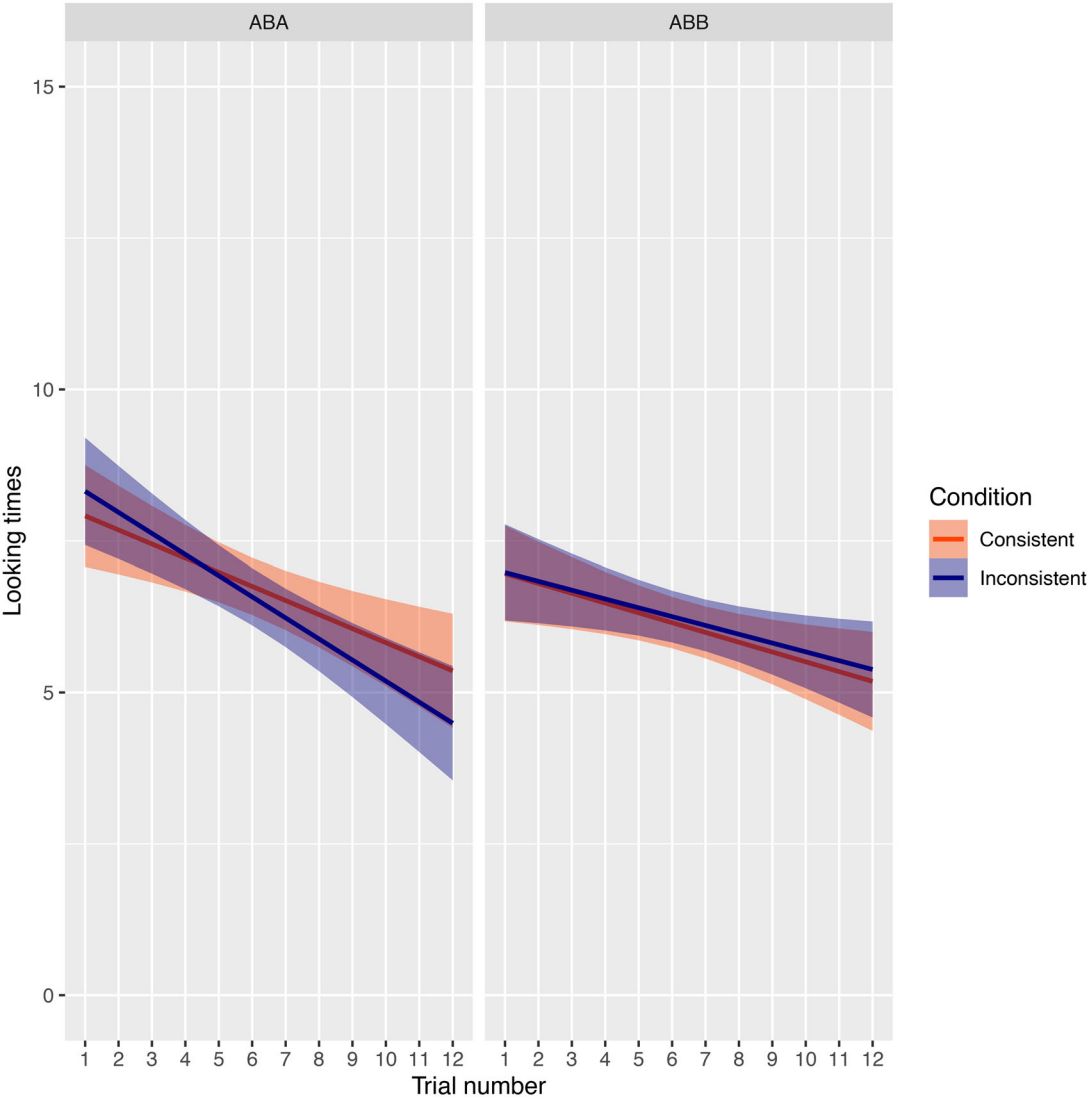
Plots of the development of looking times during the experiment for all labs combined

Next, we fitted a linear mixed‐effects model, as described in our analysis section, which allowed us to take all individual data points into account while estimating a possible learning effect. Figure 3 shows how looking times developed during the experiment. This analysis again showed no effect of test sentence consistency on looking times (*β* = 0.08, 95% CCI [−0.69, 0.84], BF_01_ = 3.08). The obtained Bayes factor indicates that these results can be interpreted as moderate evidence for the null hypothesis, which states that there is no effect of test sentence consistency on looking times. We also found moderate evidence of decreasing looking times during the experiment in general (*β* = −0.21, 95% CCI [−0.32, −0.11], BF_10_ = 9.09). Furthermore, we observed that children who heard ABB strings during familiarization looked slightly less to all test trials in general than children who heard ABA strings (*β* = −0.37, 95% CCI [−1.38, 0.68], BF_01_ = 1.36), but we cannot draw any conclusions from this outcome.

As an exploratory analysis investigating how looking times developed over the course of the experiment and to examine whether there might have been a learning trajectory during the test phase, we also ran a similar mixed‐effects model that included interaction effects between our fixed factors. This analysis showed no effect of test conditions on looking times either (*β* = 0.29, 95% CCI [−0.97, 1.33], BF_01_ = 1.70). Although the Bayes factor from this analysis is somewhat lower than from the analysis without the interaction effects, it still indicates that these results are more likely to be evidence for the null hypothesis than against it. The results for our other two main effects were comparable to those from the simple model: we found moderate evidence of decreasing looking times during the experiment in general (*β* = −0.22, 95% CCI [−0.39, −0.09], BF_10_ = 3.85), and inconclusive outcomes regarding a main effect of familiarization pattern (*β* = −0.95, 95% CCI [−2.17, −0.38], BF_01_ = 0.49). Furthermore, as in our ANOVAs, we did not see an interaction between familiarization pattern and test sentence consistency, which would have been indicative of a repetition bias (*β* = −0.44, 95% CCI [−1.86, 1.12], BF_01_ = 1.24). Crucially, we found no interaction between trial number and test sentence consistency (*β* = −0.05, 95% CCI [−0.36, 0.45], BF_01_ = 10.46), and have thus no indication of any increase or decrease of learning during the test phase of the experiment^4^.

A final comment should be made on possible lab differences. Unfortunately, as may be expected with such a large project, there are some shortcomings to the work that resulted from small technical errors discovered only at the end of the data collection. One such technical error resulted in stimuli being presented in a nonrandomized order during the test phase for all experiments conducted in the Nijmegen lab (*n = *17). In the Leiden lab, the machinery did not register any looking times for the final three trials for all participants in the ABA condition (*n *= 10). Although it is not ideal to have such missing information, given the number of babies we tested, we have sufficient statistical power to be confident about the overall patterns we observed. In addition, since in our mixed‐effects models, trial number was included as a random slope for both lab and trial type, we could take this missing information in one lab and lack of randomization in another lab into account to a certain extent. The outcomes from both these models showed evidence for the null hypothesis. It is therefore unlikely that the lack of significant differences in overall looking time is a result of any lab‐specific characteristics. Furthermore, even though average looking times for both conditions varied considerably across labs, we did not observe a difference between conditions in one of the individual labs that was close to the observed effect in the original study.

To summarize, our results do not provide any evidence in line with results from Marcus et al. (1999). We found no evidence for rule learning or rule generalization in our frequentist analyses. The additional information provided by our Bayesian analyses actually shows moderate evidence for the null hypothesis, also when taking into account the trial number. With the inclusion of the Bayesian analysis, we can therefore not only state that we do not find evidence of rule learning, but also that we have evidence that the infants we have tested were not able to use algebraic learning. Furthermore, we did not observe any interaction effects between familiarization pattern and looking times to test items, nor main effects of familiarization pattern: looking behavior and learning did not seem to depend in any way on the type of strings that children heard during the familiarization phase.

## 5 DISCUSSION

We conducted a multicenter replication of the seminal study (Marcus et al., 1999), a work that entailed a series of experiments that have inspired multiple studies extending on the original concept, and that has informed our theoretical understanding of how language and cognition are formed in the first year of life. In the more than 20 years since its publication, dozens of replication efforts and extensions have shown mixed effects, as detailed in part in a meta‐analysis by Rabagliati et al. (2019). None of these extensions have replicated the original setup as closely as the current work. We used the original stimuli and the original setup of the Headturn Preference Procedure as detailed by Marcus and colleagues, yet we were unable to replicate the original results. In fact, our Bayesian analysis actually provides evidence that the infants we tested were not able to learn and use rules algebraically. As such, these findings do not only have theoretical importance, but also highlight the added value of Bayesian analyses (Van de Schoot et al., 2014; Wagenmakers et al., 2018). The question that remains is how we can reconcile the inability to replicate the original results with the extensions using various stimuli, age groups, and species that have come since.

First, experiments showing rule extraction in older children, adults, and non‐human animals use different paradigms. With these populations, there is greater flexibility to explore and manipulate various factors that might contribute to rule‐learning abilities, such as awareness and instruction in older children and adults (Geambașu, 2018; Spit, 2022), the presence of feedback for adults (Geambașu, 2018), and extended training time and feedback for non‐human animals (Chen et al., 2015; Spierings & Ten Cate, 2016). These manipulations have all been shown to affect learner's abilities to extract and generalize rules.

The fact that there are positive results with infants in the literature, and the fact that eventually infants and young children do learn grammatical rules in natural language, which is far more complex than the simple artificial languages used here, logically presupposes that rule learning can take place at some point. Infants can eventually generalize, but they may not be able to do so under all circumstances. For example, it may be that infants of a certain age are able to learn and generalize in this manner from the original set of stimuli only if they have been exposed to English before, as was likely the case in Marcus et al. (1999)’s original set of participants recruited in the United States and tested with synthetic English stimuli. It may be the case that the English stimuli were novel to our participant group and therefore that each of the two types of patterns was equally interesting. A brief, 2‐min familiarization time with the sounds may not have been sufficient for them to overcome their focus on individual phonemes and syllables and to shift focus to structure.

There is more evidence that the effect of rule learning depends on various combinations of factors. A telling example comes from Gerken's (2006) footnote indicating that no evidence of learning occurred when testing AAB against ABB patterns, prompting a switch to the use of AAB against ABA instead, and that more robust results occurred with 9‐month‐olds, motivating the use of this age group over 7‐month‐olds in that study. While these types of failures and reasoning behind changes to the original paradigm have not been well reported, the ones that have been can offer clues from which we may better understand the degree of robustness of this effect. Rabagliati et al. (2019) have offered concrete evidence that various experimental factors might modulate rule learning. In their meta‐analysis of 63 experiments and their report on a novel experiment, they found evidence that factors such as age and meaningfulness of stimuli—regardless of whether they involve spoken language or sign language—contribute to rule learning. If the speech sounds used in our experiment were not yet meaningful for our participant group due to the nature of the sounds themselves and the brief exposure period, rule learning may not occur or biases may not prevail. A slightly longer exposure time, different stimuli, or a combination thereof, may therefore present different results.

Contrary to our expectations based on previous results in one of our own labs (Geambașu, 2018) and in the literature (e.g., Gerken, 2006; Johnson, 2009), we did not find evidence for a preference for one type of test pattern over the other, nor did we find evidence for (better) learning from one type of familiarization pattern over the other. The same explanation for the lack of overall learning offered above might apply here too; infants may have found the two types of test stimuli equally interesting because they involved non‐native speech sounds, overriding any preference for one structure over the other, regardless of any potential underlying biases for immediate repetition within such a short amount of time.

Our findings underline the importance of exact replications in the field of developmental science (Bergmann et al., 2018; The ManyBabies Consortium, 2020). Our inability to replicate the original findings despite evidence in the literature of near‐replications and extensions shows that seminal studies require repeated replication attempts and consolidation of conflicting results in the literature. The multicenter collaboration is an asset that allows us to collect more data from a larger variety of infants, thus providing us more certainty about the results than if they would be collected in a smaller sample in only one lab. A replication effort within the international ManyBabies Consortium will continue this line of work. We expect that this project, with participating labs from across the globe using a variety of paradigms, and with infants of different language backgrounds and with a large age range will allow us to better pinpoint what particular combination of factors may allow learning to occur.

## CONFLICT OF INTEREST

The authors declare that they have no conflict of interests.

## ETHICS STATEMENT

The proposed research has been approved by the Ethics Committees of each participating university in The Netherlands with the following approval codes: Amsterdam: 2020‐DP‐11840; Leiden: 2019/02; Nijmegen: ECSW/MB; Utrecht: geamb001‐01‐01‐2020.

## Data Availability

All data, stimuli, and analysis scripts are available on our OSF page: https://osf.io/hcmv7/.

## ENDNOTES

1 We report in days rather than in months;days, because the same month;day notation might refer to a different number of days, depending on the month a child is born in.

2 This whole group consisted of 52 girls and 62 boys. The mean age was 214 days (*SD =* 9 days, min–max = 196–237). Seventy‐one of these children had a monolingual background, 11 a unbalanced multilingual background, and 31 a balanced multilingual background. For one child, information on linguistic background was missing.

3 Thanks to Scott P. Johnson, who worked with Gary Marcus on subsequent extensions of the original, using the same stimuli. Gary Marcus gave permission for us to use the original stimuli as well.

4 We ran the same two models also for just the subset of monolingual children, which can be consulted on our OSF page. These models produced estimates that are comparable to the ones that are reported here, but with BFs closer to 1, which is likely because of a smaller sample size. These additional analyses do not give any reason to assume that the lack of learning in our sample has something to do with the lingual status of our participants, although it would have been difficult to make any claims about this at all, since background information about language exposure was not available in the original study.
